# Forty-two years impact of chemical fertilization on soil phosphorus partition and distribution under rice-based cropping systems

**DOI:** 10.1371/journal.pone.0305097

**Published:** 2024-06-10

**Authors:** C. Biswas, J. Ferdous, R. R. Sarker, K. R. Islam, M. M. R. Jahangir

**Affiliations:** 1 Department of Soil Science, Bangladesh Agricultural University, Mymensingh, Bangladesh; 2 Soil science Division, Bangladesh Institute of Nuclear Agriculture, Mymensingh, Bangladesh; 3 College of Food, Agricultural and Environmental sciences, The Ohio State University, Columbus, Ohio, United States of America; 4 Liebig Centre for Agroecology and Climate Impact Research, Justus Liebig University, Giessen, Germany; University of Minnesota, UNITED STATES

## Abstract

Understanding of soil phosphorus (P) transformation is crucial to minimize its edge-of-field loss associated with ecosystem disservices. A sequential chemical extraction procedure was used to assess the impact (42 years) of organic and chemical fertilizations on soil P partition and distribution under subtropical rice based cropping systems. Experimental treatments were control, N, NP, NK, NS, NZn, NPK_,_ NSZn, NPKSZn, and N+FYM (farmyard manure). Composite soils were collected from 0–5, 20–25 and 40–45 cm depths, extracted, and analyzed for soluble P, NaHCO_3_-P (inorganic and organic), NaOH-P (inorganic and organic), acid soluble (H_2_SO_4_), and residual P fractions. The NPKSZn significantly increased the concentration of soil inorganic P compared to other treatments. When FYM was applied together with N fertilizer, the organic P concentration increased, which was statistically identical to NPKSZn and NPK treatments. While the labile (NaHCO_3_-Pi, NaOH-Po), residual, and total P concentrations were stratified at 0–5 cm depth, the concentration of NaHCO_3_-Po, NaOH-Pi, and acidic P fractions increased with soil depth. The soluble, NaHCO_3_ (Pi and Po), NaOH-Pi and NaOH-Po, acidic, and residual P fractions constituted about 0.4, 6.6, 1.7, 21.3, 37.7, and 8.3%, respectively, of the total P. A higher concentration of the labile P at the surface soil indicated that the impact of chemical fertilization stratified the available P for plant uptake or susceptible to edge-of-field loss. The NPKSZn and N+FYM both had higher NaHCO_3_-Po and NaOH-Po concentrations within 40–45 cm and 0–25 cm depths, suggesting that N+FYM could promote the transformation of non-labile P into labile P pool, by reducing P fixation by soil and transport them at 20–45 cm depth. It is concluded that long-term fertilization increased the concentration of P pools especially labile P by saturating the soil adsorption sites especially in surface soil.

## Introduction

Phosphorus (P) an essential macronutrient, plays a key role to support crop productivity by regulating photosynthesis, respiration, energy storage and transfer, cell division and enlargement, root development, and grain formation [1,2]. Crop yields are often affected by low P availability in soils due to preferential adsorption and precipitation of soluble P with iron (Fe), aluminum (Al), and zinc (Zn) in acidic soils and calcium (Ca) and magnesium (Mg) in high pH soils [3]. It is reported that diverse soluble sources of P such as manures, composts, biosolids, and chemical fertilizers when applied to the soil, they are readily partitioned into available and unavailable forms, and in time react further to become highly insoluble P compounds [4].

While both Al-P and Fe-P are more abundant in acidic soils, the Ca-P dominates in neutral to alkaline soils. The Al-P and Fe-P can constitute 1 to 25% of the total soil P content [5]. The reaction between the applied P and metal oxides and/or hydroxides results in accumulation of various forms of P compounds that may not be easily accessible to plants, minimizing P-use efficiency and recovery [6]. Therefore, it is essential to apply chemical fertilizers to meet the P requirements of the crops and maintain the crop yield productivity. However, only 10 to 20% of the P fertilizers applied to the soil is used by growing crops, and the residual value rarely exceeds 50% [7], which is not an economically viable option considering the cost and resource limitations faced by farmers as it does not bring immediate economic effect. On the contrary, it can have long term effect, because phosphorus usually stays in soil as legacy P source for the future. Good strategy will be maybe therefore to create the P reserve, because the price of mineral P fertilizers will be sure increasing.

The long-term sustainability of crop productivity depends on maintaining plant available P concentration in an adequate level via application of both inorganic and organic P fertilizers or other amendments [8]. Moreover, the sustainability of agricultural production depends on developing appropriate management approaches that control the edge-of-field P loss to surface water systems while maintaining or improving crop productivity.

Long-term field experiments are important for determining and understanding crop yield trends, estimating nutrient dynamics and balances, predicting soil functional capacity, and assessing system sustainability [9]. Using different combinations of chemical fertilization under diverse cropping systems are expected to improve plant availability of P associated with its partition and distribution into different pools. However, the available information on long-term effects of different combinations of chemical fertilization on the partition and distribution of P pools in soil and its availability to crops in rice-based systems under variable soils and subtropical agro-climatic conditions are limited. In this study a sequential chemical extraction procedure was used to assess the long-term impact of organic and chemical fertilizations on soil P partition and distribution under subtropical rice-based cropping systems in dark grey silt loam soil, but the quantity of different P fractions may vary with soil type, crop type, soil management practices.

Our hypothesis is that long-term chemical fertilization will influence soil P transformation to improve its availability for supporting crop production. Using sequential chemical extractions of soil, the objective of our study was to ascertain the P partition and distribution following 42 years impact of variable combinations of chemical fertilization in rice-based cropping systems under subtropical climatic conditions.

## Materials and methods

### Site description

The study was conducted at the Bangladesh Agricultural University (BAU) experimental farm (24° 43.407°N, 90° 26.22ʹE), Mymensingh, Bangladesh. The climate is subtropical monsoon with a mean annual temperature of 26°C, average annual rainfall of 1800 mm, and relative humidity between 65 and 96% (Weather station, BAU). The soil is a dark grey silt loam Aeric Haplaquept which is non-calcareous and developed on Old Brahmaputra Floodplain sediments [10].

### Experimental design and cultural practices

**Fig 1:**
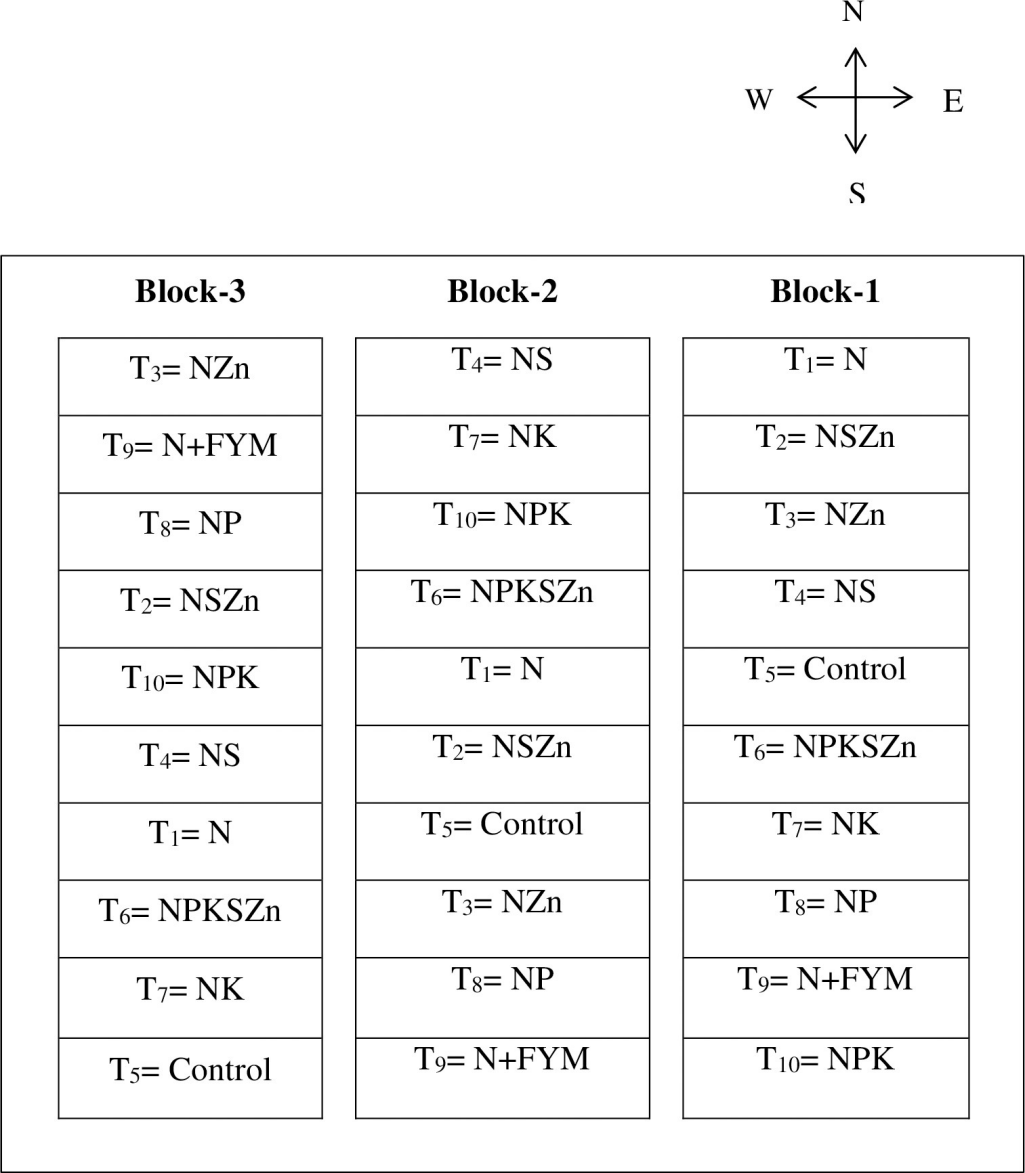
Block diagram of the experiment.

The field experiment was initiated in 1978 with an annual Boro (winter) rice–Fallow–Transplanted (T) Aman rice (monsoon) cropping system with ten different combinations of chemical fertilizers and farmyard manure (FYM) under tilled conditions. A randomized complete block design (Fig 1) with ten treatments viz., 1) control, 2) N, 3) NP, 4) NK, 5) NS, 6) NZn, 7) NPK_,_ 8) NSZn, 9) NPKSZn, and 10) N+FYM, respectively, were continued over a period of 42 yr. (Table 1). Each treatment combination was replicated thrice in field plots (12 m × 7 m = 84 m^2^). All the fertilizers except N had been applied since 1978 to all the plots as a basal dose during annual land preparation. Urea as a source of N was applied in three splits. First and second splits of urea were applied at 10 and 30 days after transplanting of rice as top dressing and final dose was applied at 50 days after transplanting. Cultural operations such as weeding, irrigation, and disease and insect control were carried out accordingly.

**Table 1:** Recommended doses of nutrients applied for growing Boro and T. Aman rice according to [11].

Nutrients	Boro rice (kg ha^-1^)	T. Aman rice (kg ha^-1^)
N	120	90
P	14	8
K	58	50
S	8	4
Zn	1	1
FYM	5 ton ha^-1^	5 ton ha^-1^

### Soil sampling and analysis

Soil cores were randomly sampled from each replicated plot after 42 crop cycles in 2022 from 0–5, 20–25 and 40–45 cm depths, respectively, using an auger and a core sampler. The soil cores collected at each depth from each replicated plot were mixed thoroughly to make composite soil samples for each replication, air-dried at room temperature in the shade (~25°C), and processed with agate mortar and pestle followed by 2 mm sieved, and stored in plastic bags until laboratory analysis.

### Phosphorus fractionation

**Fig 2:**
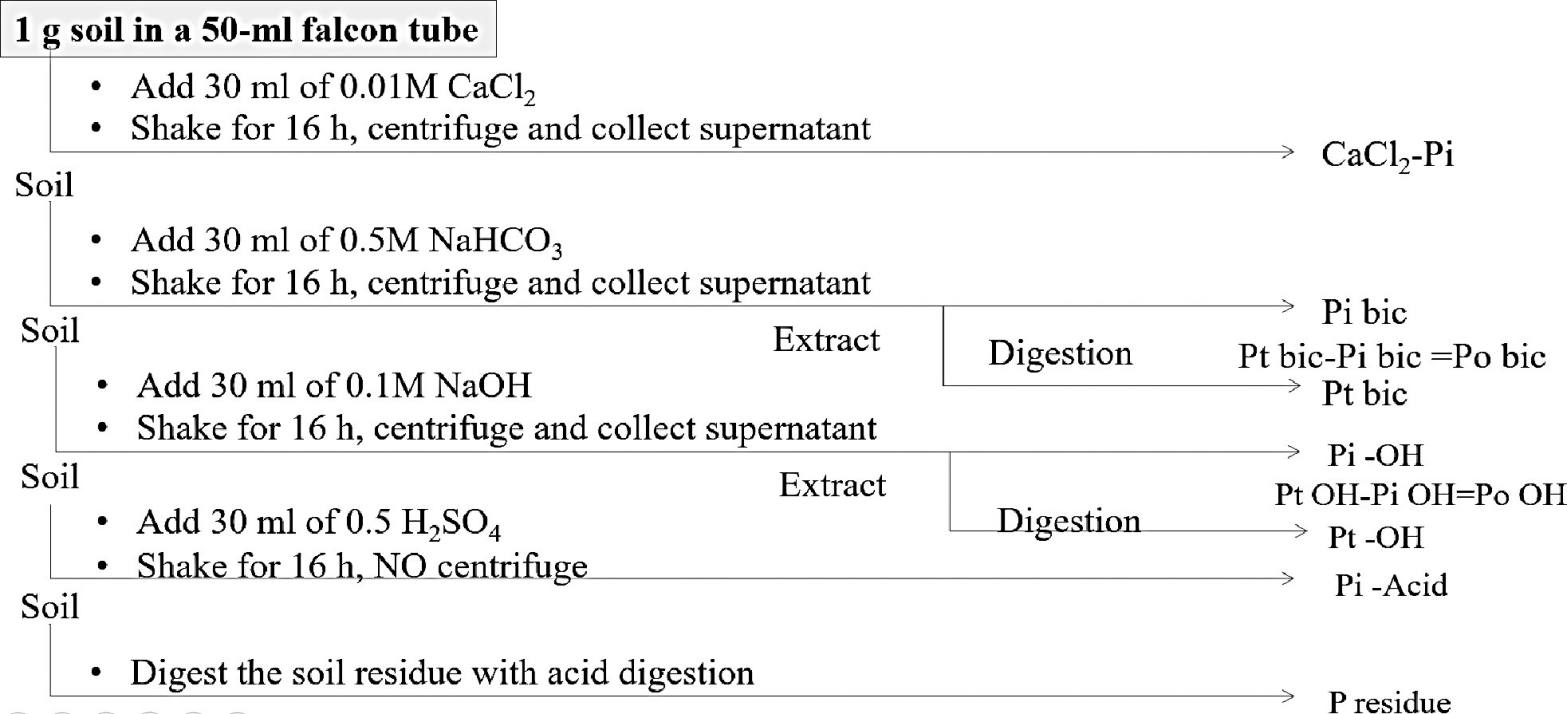
Flow chart of sequential extraction of soil P fractions.

A 1-g sample of processed soil was sequentially extracted with different chemical solutions for various soil P fractions [4,12,13]. The sequential extractions were performed as follows (Fig 2):

**Soluble P**: 0.01 M CaCl_2_ extracted inorganic P fraction.

**NaHCO**_**3**_**-P:** 0.5 M NaHCO_3_ extracted inorganic (NaHCO_3_-Pi) and organic (NaHCO_3_-Po) P pools. NaHCO_3_-Po was calculated as NaHCO_3_-Pi subtracted from the total NaHCO_3_-extractable P after digestion with H_2_SO_4_ and H_2_O_2_.

**NaOH-Pi:** 0.1 M NaOH extracted inorganic (NaOH-Pi) and organic (NaOH-Po) P pools. NaOH-Po was calculated as NaOH-Pi subtracted from the total NaOH-extractable P after digestion with H_2_SO_4_ and H_2_O_2_.

**Acid-P:** Acid P associated with negatively charged oxide surfaces through exchangeable cations and some are the occluded P. This fraction composed of insoluble and stable forms of P represent the unavailable forms of P pools in the soil. Thus 0.5 M H_2_SO_4_ was used to extract the acid P.

**Residual-P:** HNO_3_ and HClO_4_ (5: 2) extracted residual-P.

A total of seven P fractions, including soluble P, NaHCO_3_-P (Pi and Po), NaOH-P (Pi and Po), acid P, and residual P were extracted. The ammonium molybdate-ascorbic acid method [14] was used to measure the concentration of P in the extracts and digests following neutralization (when needed) with 0.9M H_2_SO_4_. The absorbance of P was measured spectrophotometrically at 882 nm. Following the same digestion procedure to determine residue P, the total P was determined.

### Statistical analysis

Multivariate statistical analyses to evaluate whether the concentration of different P pools in soils as impacted by the variable chemical fertilization and FYM amendments were evaluated by a two-way analysis of variance (ANOVA) using Statistix10. While fertilization and soil depth were considered as fixed predictor variables, the block was considered as a random predictor variable. Main effects of fertilization, soil depth and their interaction were determined using Fisher’s Least Significant Difference (LSD) Test at p≤0.05 level, unless otherwise mentioned. The Pearson correlation coefficient comparison test was performed to establish relationships among extracted P fractions in soil.

## Results

**Fig 3:**
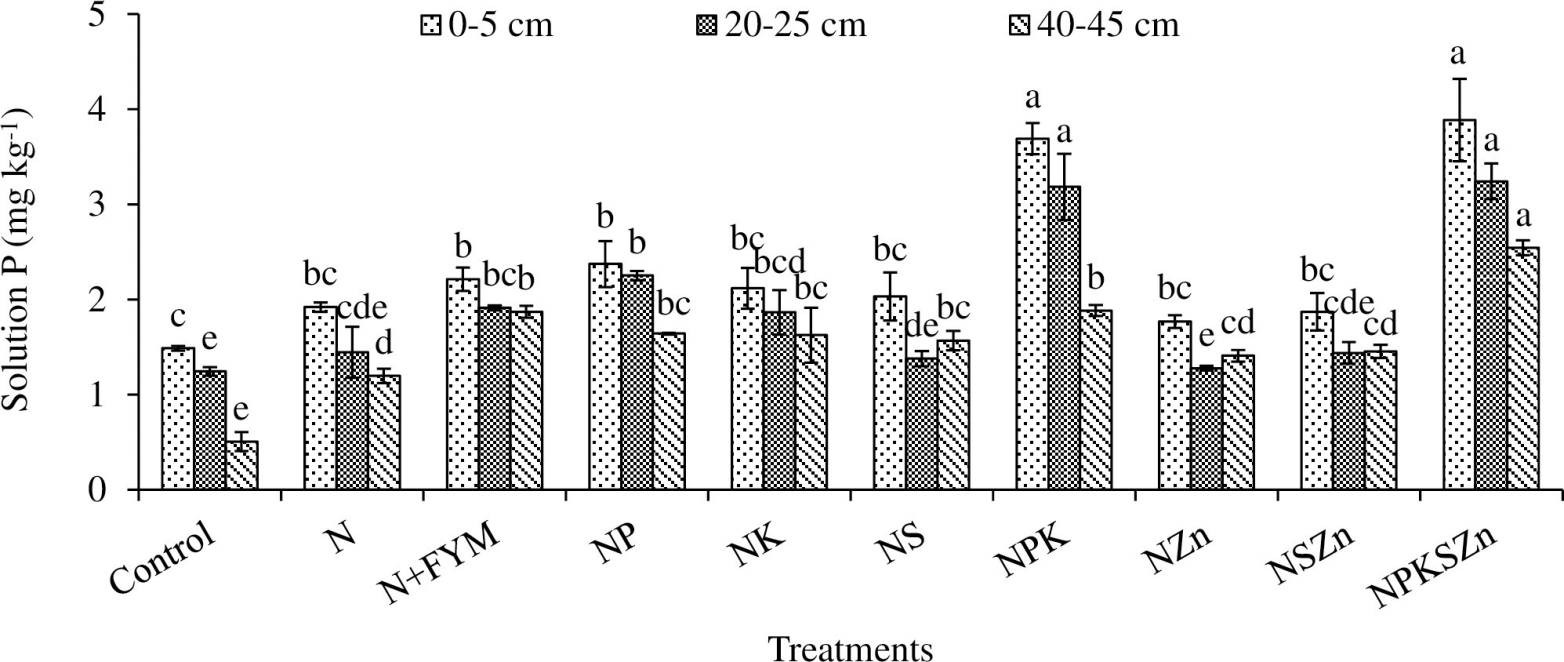
Long-term effects of chemical fertilization and farmyard manure amendments on soluble P concentration at different soil depths. Bar (mean ± standard error) with different letters vary significantly (p ≤ 0.05) to each other at same depth for different treatments.

**Fig 4:**
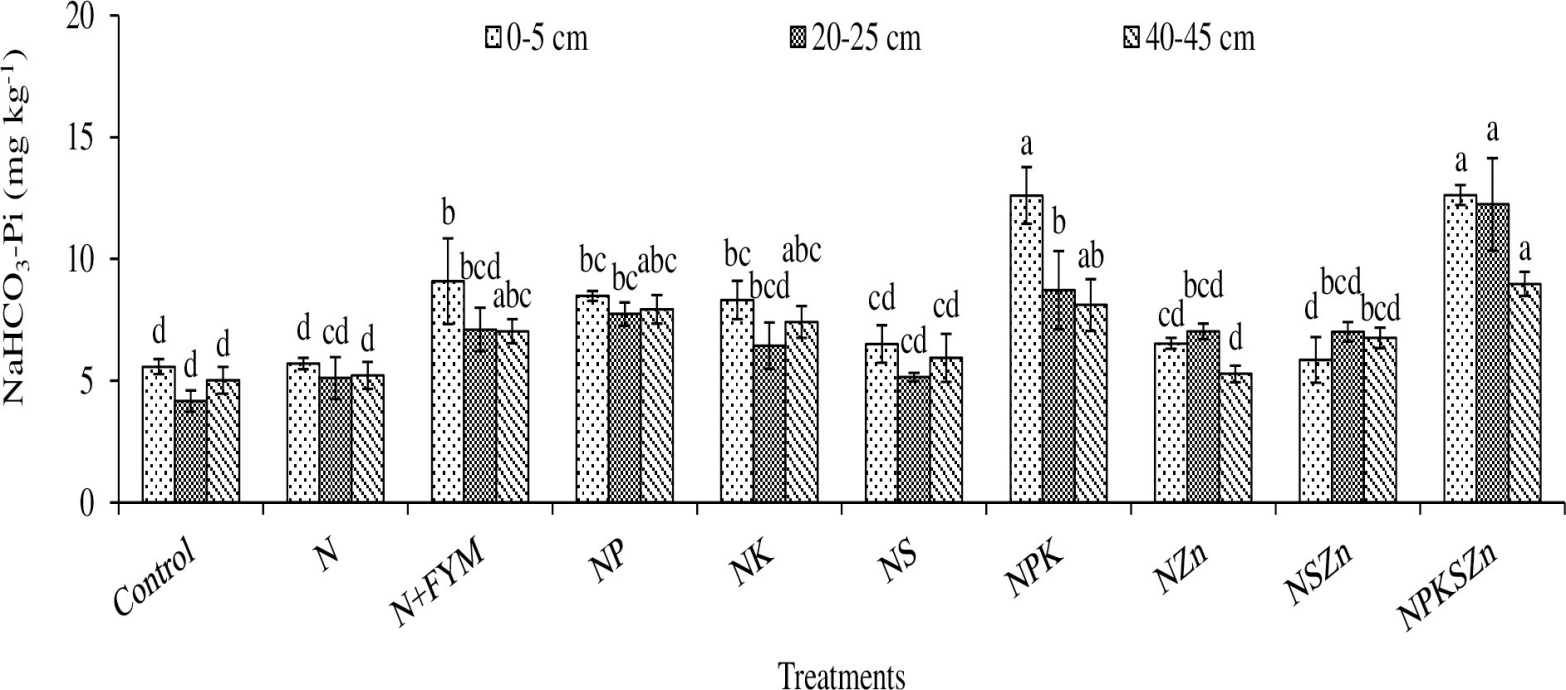
Long-term effects of chemical fertilization and farmyard manure amendments on NaHCO_3_-P_i_ concentration at different soil depths. Bar (mean ± standard error) with different letters vary significantly (p ≤ 0.05) to each other at same depth for different treatments.

**Fig 5:**
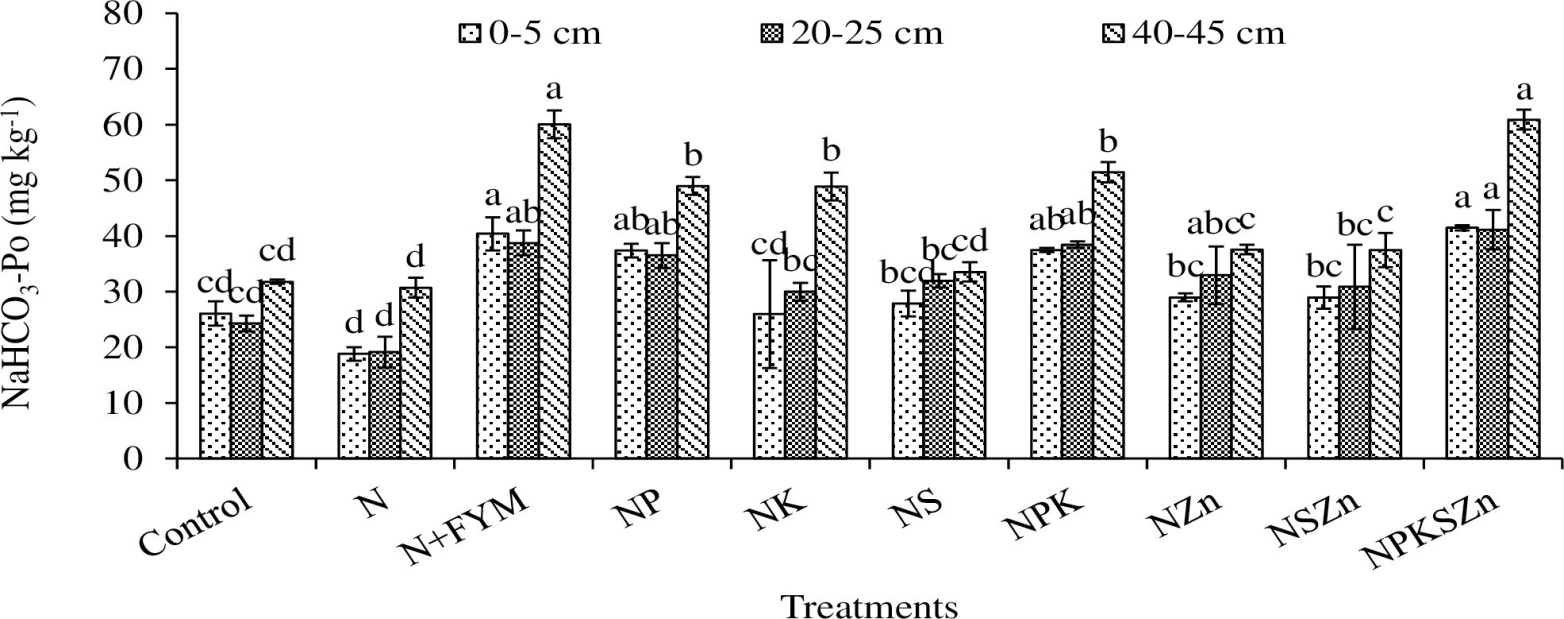
Long-term effects of chemical fertilization and farmyard manure amendments on NaHCO_3_-Po concentration at different soil depths. Bar (mean ± standard error) with different letters vary significantly (p ≤ 0.05) to each other at same depth for different treatments.

**Fig 6:**
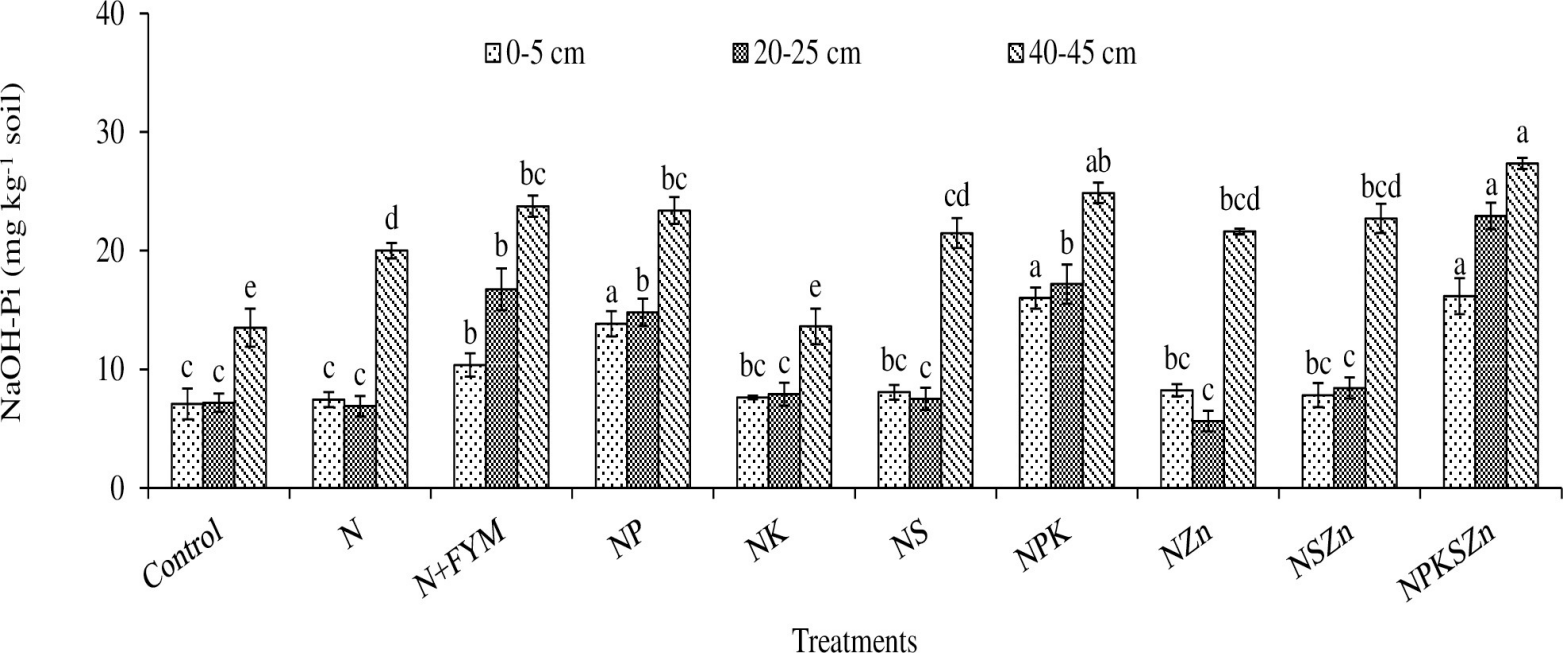
Long-term effects of chemical fertilization and farmyard manure amendments on NaOH-Pi concentration at different soil depths. Bar (mean ± standard error) with different letters vary significantly (p ≤ 0.05) to each other at same depth for different treatments.

**Fig 7:**
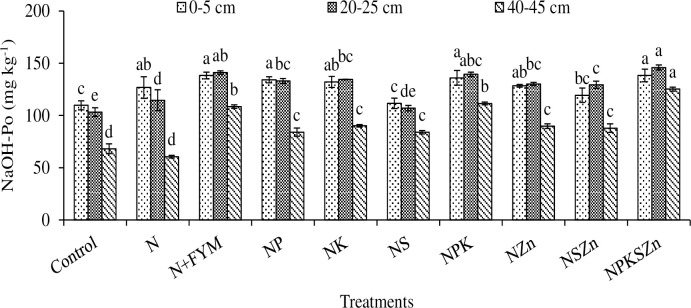
Long-term effects of chemical fertilization and farmyard manure amendments on NaOH-P_o_ concentration at different soil depths. Bar (mean ± standard error) with different letters vary significantly (p ≤ 0.05) to each other at same depth for different treatments.

**Fig 8:**
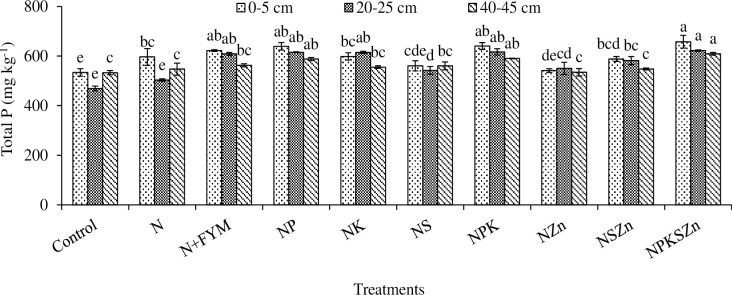
Long-term effects of chemical fertilization and farmyard manure amendments on total P concentration at different soil depths. Bar (mean ± standard error) with different letters vary significantly (p ≤ 0.05) to each other at same depth for different treatments.

The long-term chemical fertilization and farmyard manure (FYM) amendments have impacted significant changes in soluble P, NaHCO_3_ Pi, NaHCO_3_ Po, NaOH Pi, NaOH Po, acidic P, residual P, and total P concentrations (Figs 3–8 and Tables 2–4).

**Table 2:** Long-term effects of chemical fertilization on acid and residual phosphorus concentration (mg kg^-1^) at different soil depths (mean ± standard error of mean).

	0–5 cm		20–25 cm		40–45 cm	
Treatments	Acid-P	Residual P	Acid-P	Residual P	Acid-P	Residual P
Control	201.0±9.4c	33.5±2.6c	207.6±10.8b	39.2±0.8c	216.6±19.2c	39.3±0.9c
N	207.4±12.9c	53.0±0.8ab	242.9±46.2ab	38.9±1.8bc	254.0±7.5abc	44.7±2.0bc
NSZn	216.4±17.1bc	50.7±0.4ab	247.4±23.4ab	39.9±1.8c	251.7±2.1ab	40.6±2.6bc
NZn	222.7±9.1abc	45.5±1.6b	237.5±13.3ab	39.5±0.9bc	233.5±20.5bc	45.9±2.8b
NS	223.7±2.3abc	46.1±8.0ab	210.1±6.3ab	43.6±1.2ab	240.7±18.6bc	52.9±1.0a
NPKSZn	247.2±10.9a	57.1±6.1a	259.6±9.2a	46.0±1.0a	264.2±20.4bc	57.2±2.6a
NK	222.2±15.9abc	47.1±2.4ab	249.1±13.2ab	44.3±1.0a	239.9±6.0bc	43.5±0.7bc
NP	236.3±4.9ab	55.6±2.9ab	233.5±9.0ab	44.1±2.0ab	249.6±13.9bc	53.4±2.5a
N+FYM	235.0±5.4ab	53.9±1.1ab	257.4±0.7ab	44.5±1.4a	251.2±10.9abc	54.0±1.9a
NPK	241.3±4.1ab	56.2±4.4ab	249.8±4.7ab	45.1±1.6a	292.7±7.5a	53.4±2.2a
CV (%)	6.5	13.4	12.6	6.5	10	7.3
*Level of significance*	*	*	ns	**	ns	***

CV (%) = coefficient of variations. LOS = Level of significance

*, **, *** indicates p ≤ 0.05, p ≤ 0.01, p ≤ 0.001, respectively. Means separated by same letter at each column were not significantly different for the treatments.

**Table 3:** Long-term effects of chemical fertilization and farmyard manure amendments on relative concentrations (%) of different P fractions at 0–5, 20–25 and 40–45 cm soil depth.

Depth (cm)	Solution P (%)	NaHCO_3_-Pi (%)	NaHCO_3_-P_o_ (%)		NaHCO_3_-P (%)	NaOH-Pi (%)	NaOH-P_o_ (%)	NaOH-P (%)	Acid P (%)	Residual P (%)
0–5	0.39a	1.34a		5.21b	6.56b	1.69b	21.33a	23.02a	37.75b	8.31a
20–25	0.33ab	1.22a		5.62b	6.84b	1.97b	22.31a	24.28a	42.85a	7.46b
40–45	0.28b	1.21a		7.80a	9.00a	3.76a	16.10b	19.86b	44.30a	8.60a

Here, Pi = inorganic phosphorus fraction, Po = organic phosphorus fraction, P = Total (Organic and inorganic phosphorus fraction), CV (%) = coefficient of variations. LOS = Level of significance and

*, **, *** indicates p ≤ 0.05, p ≤ 0.01, p ≤ 0.001, respectively. Means separated by same letter at each column were not significantly different for the treatments.

**Table 4:** Correlation coefficient values of the relationship among different P fractions.

	Solution P	NaHCO_3_-_Pi_	NaHCO_3_-Po	NaOH-Pi	NaOH-Po	Acid P	Residual P
NaHCO_3_-Pi	0.82***						
NaHCO_3_-Po	0.54**	0.57***					
NaOH-Pi	0.79***	0.75***	0.68***				
NaOH-Po	0.50**	0.52**	0.46*	0.41*			
Acidic P	0.59***	0.64***	0.62***	0.56**	0.44*		
Residual P	0.51**	0.43*	0.37*	0.44*	0.68***	0.40*	
Total P	0.64***	0.65***	0.44*	0.67***	0.43*	0.55**	0.39*

Here, Pi = inorganic phosphorus fraction, Po = organic phosphorus fraction, P = Total (Organic and inorganic phosphorus fraction)

*p < 0.05

**p < 0.01

***p < 0.001, ns = not significant. Means separated by same letter under each column were not significantly different at p ≤ 0.05 among the treatments.

### Soluble P

Chemical fertilization and N+FYM amendments significantly impacted soluble P concentration at all soil depths (Fig 3). The maximum concentration of soluble P (3.9, 3.2, and 2.5 mg kg^-1^) was recorded in NPKSZn treatment, which was statistically alike NPK in the upper two soil depths, and was followed by NP, N+FYM, NK, NS, N, NSZn, and NZn, where all seven treatments were statistically identical among themselves. In contrast, the control had the lowest concentration of soluble P (1.5, 1.2, and 0.5 mg kg^-1^) at all depths. Compared to the control, the soluble P concentration increased by 161% in NPKSZn, but only by 49–54% in N+FYM. As expected, soluble P concentration decreased with soil depth (Fig 3).

### NaHCO_3_-Pi

Alike to soluble P, the NaHCO_3_-Pi concentration significantly influenced by chemical fertilization and N+FYM amendments at all soil depths (Fig 4). The higher concentration of NaHCO_3_-Pi (12.6, 12.2 and 9 mg kg^-1^ at 0–5, 20–25 and 40–45 cm depths) was obtained in NPKSZn that was statistically similar to NPK followed by N+FYM, NP, and NK, respectively. In contrast, lower concentration of NaHCO_3_-Pi was extracted in the control (5.6, 4.2 and 5 mg kg^-1^) which was statistically identical to N, NSZn, NZn and NS treatments at 0–5 and 40–45 cm depths. The NaHCO_3_-Pi concentration was higher in NPKSZn by 126, 194, and 79%, whereas it was only 63, 71, and 53% in N+FYM at 0–5, 20–25, and 40–45 cm depth, respectively, over the control. Unlike the soluble P, the concentration of NaHCO_3_-Pi increased with soil depth (Fig 4).

### NaHCO_3_-Po

Long-term effects of chemical fertilization and N+FYM amendments significantly affected the NaHCO_3_-Po concentration at all soil depths (Fig 5). The NPKSZn had the highest concentration of NaHCO_3_-Po at 0–5, 20–25, and 40–45 cm depths, respectively. This was statistically similar to NP, N+FYM, and NPK treatments at 0–5 cm depth, NZn, NP, N+FYM, and NPK treatments at 20–25 cm and 40–45 cm depths. In contrast, a lower concentration of NaHCO_3_-Po (18.8, 19.1 and 30.7 mg kg^-1^ at 0–5, 20–25 and 40–45 cm, respectively) was observed in N treatment alone that was statistically similar to NS, control, NK at 0–5 and 20–25 cm depths, and NS and control at 40–45 cm depth. The NaHCO_3_-P_o_ increased by 120, 115, and 98% at 0–5, 20–25 and 40–45 cm depths, respectively, in NPKSZn whereas increased by 115, 102 and 96% in N+FYM over the control at 0–5, 20–25 and 40–45 cm depth, respectively. Except for NSZn, the concentration of NaCO_3_-Pi decreased with soil depth (Fig 5).

### NaOH-Pi

The maximum concentration of NaOH-Pi (16.2 mg kg^-1^ at 0–5 cm, 22.9 mg kg^-1^ at 20–25 cm and 27.3 mg kg^-1^ at 40–45 cm depth) was recorded in NPKSZn (Fig 6). However, the N alone treatment had the lowest NaOH-Pi concentration including 7.1 mg kg^–1^ at 0–5 cm and 13.5 mg kg^–1^ at 40–45 cm depths, whereas the NZn had the lowest concentration of 5.6 mg kg^–1^ at 20–25 cm depth. A slight increase in NaOH-Pi concentration was observed at 40–45 cm depth which ranged from 13.5 to 27.3 mg kg^-1^. The concentration of NaOH-Pi increased over the control by 128, 306, and 102% in NPKSZn at 0–5, 20–25, and 40–45 cm depths, respectively, whereas it increased by 46, 197, and 76%, respectively in N+FYM. Except for N, NS and NZn treatments, the concentration of NaOH-Pi increased with soil depth.

### NaOH-Po

When chemical fertilizer and N+FYM were applied separately or together, the NaOH-Po was significantly affected at all soil depths (Fig 7). The highest concentration of NaOH-Po 138.4, 145.9 and 125.2 mg kg^-1^ was determined in NPKSZn at 0–5, 20–25 and 40–45 cm depths, respectively. At 0–5 cm depth, the concentration of NaOH-Po ranged between 109.7 to 138.4 mg kg^-1^ and the maximum concentration was statistically alike NP, N+FYM and NPK. The range of NaOH-Po concentration was 103.2–145.9 mg kg^-1^ at 20–25 cm depth; the highest concentration in NPKSZn was statistically alike N+FYM and NPK. The NaOH-Po ranged from 60.6 to 128.6 mg kg^-1^ at 40–45 cm of depth, showing a small reduction; the lowest value was recorded in N alone treatment. At 0–5, 20–25, and 40–45 cm depths, the NaOH-Po concentration increased over the control by 26, 41, and 107% in NPKSZn, and 26, 37, and 79% in N+FYM. With the increase in soil depth, the NaOH-Po concentration reduced while within 20–25 cm, the concentration was higher than other depths.

### Acid P

The sole or combined application of chemical fertilization and N+FYM amendments had a significant impact on acid-P at 0–5 cm depth whereas non-significant response was observed at 20–25 and 40–45 cm depths (Table 2). At 0–5 cm depth, acid-P ranged from 201 to 247.2 mg kg^-1^ with maximum concentration of 292.7 mg P kg^-1^ was observed in NPK at 40–45 cm depth. In contrast, the control had the lowest concentration of acid-P (201 mg kg^-1^), which was statistically alike N_,_ NSZn and NZn, NS and NK treatments. The NPKSZn had the highest increase in acid-P, when compared to the N+FYM over the control. Acid-P was similar within the soil depths under different treatments.

### Residual P

The residual P concentration ranged from 33.4 to 57 mg kg^-1^ at 0 to 5 cm depth and the highest residual P concentration was observed in NPKSZn, which was statistically alike with other treatments except NZn and the control (Table 2). Residual P concentration at 20–25 cm depth varied from 37.2 to 46 mg kg^-1^ and the maximum concentration was found in NPKSZn, whereas minimum concentration was recorded in the control that was statistically identical to NSZn, NZn and control treatments. A slight increase in residual P concentration was found at 40–45 cm depth which ranged from 39.3 to 57.2 mg kg^-1^. The highest increase in residual P concentration (71, 18 and 45% at 0–5, 20–25 and 40–45 cm depth, respectively) was found in NPKSZn whereas the increase was observed by 61, 14 and 37% in N+FYM at 0–5, 20–25 and 40–45 cm depths, respectively, over the control.

### Total P

Total P concentration was highest in NPKSZn (657.5, 621.8, and 608.7 mg kg^-1^) and lowest in the control (533.9, 469, and 532.8 mg kg^-1^) at all soil depths (Fig 8). At 0–5 cm depth, total P concentration varied from 533.9 to 657.5 mg kg^-1^ in NPKSZn which were statistically alike NP_,_ N+FYM and NPK treatments whereas the control, in contrast, was statistically alike to NZn and NS. A slight decrease in total P concentration was obtained at 20–25 cm depth which ranged from 469 to 621.8 mg kg^-1^. At 20–25 cm depth, the total P concentration in NPKSZn was statistically like NK, NP, N+FYM and NPK whereas the control was statistically identical to N alone treatment. At 40–45 cm depth, total P concentration ranged from 532.8 to 608.7 mg kg^-1^. Total P concentration increased by 23, 33 and 14% in NPKSZn whereas it was 16, 30 and 6% in N+FYM over the control at 0–5, 20–25 and 40–45 cm depths, respectively. Total P concentration was higher in the upper soil depth than in the lower depth (Fig 8).

### Relative concentration of the extracted phosphorous pools

The data of Table 3 indicated that the reserve of the recalcitrant P pools in soil i.e., organic NaOH-P and residual P might have retained over decades of rice production without proper P management. Among the P fractions, the acid-P contributed highly to total P (37–44%) within 0–45 cm soil depth which was followed by NaOH in both organic (Po) and inorganic (Pi) forms. The mean of the treatments at different soil depths showed that solution and NaOH-P accounted for 0.4% and 20–24% of the total P, which were higher within 0–25 depth; however, the percentage reduced for NaHCO_3_-P pols. There were significant linear and positive correlations (r = 0.37 to 0.82) among soluble P, NaHCO_3_-Pi, NaHCO_3_-Po, NaOH-Pi, NaOH-Po, acid-P, residue P, and total P concentrations (Table 4). The NaHCO_3_-Pi significantly correlated with the soluble P (r = 0.82) accounting 67% of the variability in soluble P concentration. A significant linear and positive correlation between NaHCO_3_-Pi and NaHCO_3_-Po was also found (r = 0.57). The NaOH-Pi moderately correlated with NaOH-Po (r = 0.41). Total P and all other P fractions similarly showed positive linear and significant correlations among themselves (Table 4).

## Discussion

### Soluble P

A significantly higher concentration of soluble P under NPKSZn and NPK compared to other treatments was attributed due to P stratification in response to the long-term effects of 42-yr. of chemical fertilization [15]. While the P adsorption sites are gradually saturated over time, the soluble P in post-saturated soils is weakly adsorbed and consequently increases P availability to crops [16] or susceptible to edge-of-field loss via runoff and drainage [2] (Rahman et al., 2021). As expected, the FYM application did not increase the soluble P, which was collaborated with the results of previous studies [17]. It is reported that manuring P did not increase the soluble P concentration as much as by inorganic P fertilization [17], which indicates the presence of large amount of inorganic P than organic P compounds in the soil.

A significant decrease in soluble P concentration with increasing soil depth is well-corresponded with results of Rajeswar et al. [18], who reported that solution P decreased gradually from the surface to the subsurface depths due to the effects of P fixation with soil reactive components like Al, Fe, and Ca oxides and hydroxides.

### NaHCO_3_-Pi

NaHCO_3_-Pi, considered as inorganic labile P, is a biologically available form of P [19]. The NaHCO_3_-Pi concentration was higher in NPKSZn and NPK treatments, indicates that use of phosphate fertilizer significantly partitioned to increase the inorganic forms of P [20]. The N+FYM amended soil had higher NaHCO_3_-Pi concentration alike other P treatments, suggesting that organic manure added inorganic P which contributed to the NaHCO_3_-Pi, where FYM competes with the adsorption sites associated with the P fixation in soil [21,22]. The primary form of P (54–85%) in manures is inorganic, which justifies that the organic amendments could increase inorganic labile P (NaHCO_3_-Pi) in soil for plant uptake [23]. The concentration of NaHCO_3_-Pi was lower at subsurface depth than that at the surface depth, indicating that the amorphous Al and Fe could bind the NaHCO_3_-Pi. Long-term P fertilization of conventionally-tilled soils provided more surface contacts between P and the reactive components that increased availability of inorganic labile P in the surface soil. The higher NaHCO_3_-Pi concentration in the slightly acidic surface soil was most likely associated with the fixation of inorganic P with organic compounds and Fe, Al, and Ca oxides and hydroxides in response to long-term chemical fertilization and leaching of basic cations in partially waterlogged soil environment under rice production [24].

### NaHCO_3_-Po

The NaHCO_3_-Po is considered an easily mineralizable organic P that increased with soil depth unlike inorganic P forms. Under conventionally tilled system, the surface soil is warm and aerobic which causes an oxidation of soil organic matter compared to the partially anaerobic subsoil [25] due to limited O_2_ diffusion rates. The NPKSZn and N+FYM both had similar NaHCO_3_-Po concentration within 40–45 cm depth attributed due to mineralization of NaHCO_3_-Po and in organic sources the P is present mostly in inorganic form. The antagonistic effect of simultaneous P and Zn application might be absent due to the low nutrient status in the experiment field and the added nutrients were used by plants for their growth. Long-term application of FYM also provides organic acids which compete and reduce the P adsorption sites and strength to soil particles, thus influences the downward movement within soils. Our result indicates that higher mineralization of organic sources of P causes saturation of adsorption sites of surface soil, thus the NaHCO_3_-Po moved downward and accumulated.

### NaOH-Pi

The inorganic fraction of NaOH extracted P (NaOH-Pi) is strongly adsorbed onto Fe, Al and clay minerals [12] and mostly contributes to long term P transformation. However, the concentration of NaOH-Pi varied significantly being higher in NPKSZn compared to other treatments showed a positive relationship with soil depth because of adsorption of P by clay minerals, Fe, and Al oxides and hydroxides within illuvial zones [12,26]. Likewise, Coelho et al. [27] reported that annual P fertilization increased the concentration of NaOH-Pi at both 0–20 and 20–40 cm depths. The adsorption capabilities of NaOH-Pi, which preferentially reacted with Al, Fe, and clay minerals in the subsurface depth, can be used to explain the concentration trend that rises with soil depth.

### NaOH-Po

Alike NaHCO_3_-Po, the NaOH extracted organic P (Po) is associated with fulvic and humic acids adsorbed onto mineral and SOM surfaces [28]. Our results are in agreement with the results of Conte et al. [29], who reported a high concentration of NaOH-Po in long-term P fertilized soils. While Verma et al. [30] observed that NaOH-Po content increased with the application of 100% NPK+FYM, it decreases in the control. A significantly higher concentration of NaOH-Po than that of the NaHCO_3_-Po agreed with the results of Guo et al. [31]. An increasing NaOH-Po concentration in soils under long-term chemical fertilization i.e., NPKSZn compared to the unfertilized ones could be the result of continuous and cumulative amount of root biomass being added to the soil over time. The mineral and organic fertilization and/or amendments both acts as P sources [32] to increase the concentration of moderately stable to stable NaOH-Po at 0–25 cm depth, facilitated by continuous tillage in each of the rice growing seasons. This might be a result of the saturation of P sites in upper soil layers and the excess P moved to the sub-surface. It further indicates that stable NaOH-Po may acts as a potential source of P in case of P deficient soils, similar result was also reported in previous study [33]. The NaOH-Po may move downward by forming Al-P complex as a potential source at 20–25 cm depth, similar result is documented [34]. Ahmed et al. [35] reported that greater levels of organic P in the soil surface are usually positive for plant absorption.

### Acidic and residual P

A significantly higher concentration of acid-P at 40–45 cm depth was due to greater impact of the chemical fertilization (NPK), when compared to the other depths. In contrast, Saleque et al. [4] obtained dissimilar findings that the long-term application of both inorganic and organic P significantly increased acid-P at 0–15 cm depth. Soil exchange sites are generally saturated by P in response to long-term fertilization and excess P moved to sub-soils where they were accumulated and fixed with clay minerals. Acid-P movement and accumulation in sub-soils can be a natural mechanism of P sequestration in soil profile. According to Saleque and Kirk [36], rice plants can still absorb P from the acid-P fraction in lowland conditions via biochemical pathways. The acid-P pool accounted for 25% of the total P uptake by rice. Accumulation of residual P could be associated with greater amount of P adsorbed by soil reactive compounds into unavailable forms and accumulated over time by the impact of chemical fertilization. While soil reactive compounds can fix soluble P before crops uptake [37–39], in contrast, indicated that P fertilization had minimal effect on both organic and residual P fractions but mostly supplied soluble inorganic P to crops.

### Total P

Total P content at different soil depths responded positively due to the temporal effects of long-term chemical fertilization. Dutta et al. [40] reported that total P content was significantly higher under NPK fertilization when integrated with organic amendments (FYM) compared to only chemical fertilization. However, an opposite trend was observed in our study as N+FYM was unable to fulfil the requirements of P fertilization for crops because application of FYM together with mineral nitrogen improved the mineralization and subsequently the release of P into the available forms. Again, the rate of FYM application may not be sufficient to increase P content in soil since the soil was nutrient poor initially. These available forms of nutrients might be taken up with plants and probably FYM supplied low P content thus the P content in soil decreased compared to other treatments except control. This study indicates that only organic source of nutrient was not sufficient to supply all the essential plant nutrients, especially P to increase total P content in soil.

### Relationship among soil P fractions

Significant positive and linear correlation (Pearson) of soluble and labile P with NaOH extracted P (Pi and Po) was due to the effects of weak association of P with Fe or Al and their oxides, clay minerals, humic acid, or fulvic acid. This can be explained by the saturation of adsorption sites of Fe and Al and their oxides due to long-term chemical P fertilization. The soluble P applied through inorganic and organic fertilizers cannot be fixed by the adsorption sites and remain available to crops once it reaches adsorption saturation. The saturation of the adsorption sites can also explain how certain P fractions correlate with one another.

## Conclusions

Long-term chemical fertilization significantly impacted the partition and distribution of P in different pools with acid-P was the dominant fraction. Most of the P fractions at different soil depths increased significantly over the control via entirely chemical fertilization over the years. The level of easily available organic P (NaHCO_3_-P_o_) increased at subsurface soil irrespective to fertilization effect, but the inorganic P increased at surface soil. Long-term chemical fertilization under repeated ploughing may be responsible for high decomposition of SOM at surface depth which resulted in higher available Pi at 0–5 cm depth and accumulation of Po at lower depths (20–45 cm). The higher concentration of easily available forms of P at surface soil indicated that both organic and inorganic sources provide available P while other inorganic sources (N-K) positively influenced the P availability. The higher labile P forms content in surface soil indicated that supplied P enriched the plant available P pool. The NPKSZn and N+FYM both had higher NaHCO_3_-Po and NaOH-Po within 40–45 cm and 0–25 cm depth, respectively, indicating that FYM could promote the transformation of non-labile into more labile P forms, with an associated reduction in P fixation by soil particles and translocate them at 20–45 cm depth.
